# Bruch's membrane opening minimum rim width and retinal nerve fiber layer thickness in a Brazilian population of healthy subjects

**DOI:** 10.1371/journal.pone.0206887

**Published:** 2018-12-18

**Authors:** Camila S. Zangalli, Jayme R. Vianna, Alexandre S. C. Reis, Jamil Miguel-Neto, Claude F. Burgoyne, Balwantray C. Chauhan, Vital P. Costa

**Affiliations:** 1 Department of Ophthalmology, University of Campinas, Campinas, Brazil; 2 Department of Ophthalmology and Visual Sciences, Dalhousie University, Halifax, NS, Canada; 3 Optic Nerve Head Research Laboratory, Devers Eye Institute, Portland, OR, United States of America; Bascom Palmer Eye Institute, UNITED STATES

## Abstract

**Objective:**

To determine Bruch’s membrane opening (BMO) minimum rim width (MRW) and peripapillary retinal nerve fiber layer thickness (RNFLT) measurements, acquired with optical coherence tomography (OCT) in healthy Brazilian individuals self-reported as African Descent (AD), European Descent (ED) and Mixed Descent (MD).

**Methods:**

260 healthy individuals (78 AD, 103 ED and 79 MD) were included in this cross-sectional study conducted at the Clinics Hospital of the University of Campinas. We obtained optic nerve head (24 radial B scans) and peripapillary retinal nerve fiber layer (3.5-mm circle scan) images in one randomly selected eye of each subject.

**Results:**

After adjustment for BMO area and age, there were no significant differences in mean global MRW (P = 0.63) or RNFLT (P = 0.07) among the three groups. Regionally, there were no significant differences in either MRW or RNFLT in most sectors, except in the superonasal sector, in which both MRW and RNFLT were thinner among ED (P = 0.04, P<0.001, respectively). RNFLT was also thinner in ED in the inferonasal sector (P = 0.009). In all races, global MRW decreased and global RNFLT increased with BMO area. AD subjects had higher rates of global RNFLT decay with age (-0.32 μm/year) compared to ED and MD subjects (-0.10 μm/year and -0.08 μm/year, respectively; P = 0.01 and P = 0.02, respectively).

**Conclusions and relevance:**

While we found no significant differences in global MRW and RNFLT among the three races, age-related thinning of the RNFLT was significantly higher in the AD subgroup, which warrants further study.

## Introduction

Assessment of the optic nerve head (ONH) is vital for the detection and follow-up of glaucoma damage. Conventionally, clinical [1] and instrument-based [2–6] evaluation of the ONH estimate the ONH neuroretinal rim in the plane of the visible optic disc margin. However, using optical coherence tomography (OCT), a series of studies have shown that the disc margin is not a consistent OCT anatomic landmark contributing to errors in rim parameters, and that for OCT rim assessment, Bruch’s membrane opening (BMO) is a more suitable landmark [7, 8]. OCT BMO Minimum Rim Width (MRW) is a parameter that uses BMO as the anatomic reference point and MRW is defined as the shortest distance between BMO and the internal limiting membrane, in effect, measuring the neuroretinal rim perpendicular to the trajectory of the overlying rim tissue [9, 10]. It therefore takes into account the orientation of the RGC axons as they pass through BMO to enter the anterior neural canal. MRW measurements have demonstrated better diagnostic performance for detecting glaucoma than other rim parameters [11] and better structure-function relationships [12] with visual field testing.

In addition to this new parameter, acquisition and regionalization of data is now based on the subject’s individual fovea to BMO center (FoBMO) axis, which is highly variable among subjects [13, 14]. ONH phenotyping with MRW has recently been described in Caucasians [15, 16] and African Americans [16] from the US and Europe. However, ONH phenotypes may vary considerably in healthy subjects across other racial groups. Marsh and colleagues have shown that individuals of European Descent (ED) have significantly smaller optic disc areas (based on the clinically visible optic disc margin) than Hispanics and African Americans [17]. Similarly, Girkin and colleagues found that African Descents (AD) have larger optic discs areas compared to ED [18]. The same authors also showed that retinal nerve fiber layer thickness (RNFLT) varies by quadrant across racial groups: ED individuals had the thinnest RNFLT measurements except in the temporal quadrant, corresponding to the papillomacular bundle, which was thinner in the AD group [18].

The majority of the Brazilian population is the product of three ancestral roots: Europeans, Africans, and Amerindians and miscegenation among these groups is highly prevalent [19]. Furthermore, ADs from Brazil have emigrated from different regions of Africa compared to those who migrated to the US.[20–22] Therefore, it may well be that the ONH phenotypic characteristics of the Brazilian population may not be comparable to those of other countries and may differ from OCT databases currently being used. The purpose of this study was to determine MRW and RNFLT measurements in a normal Brazilian population of self-reported AD, ED and MD (individuals who reported both European and African ancestry). In addition, we evaluated the effects of age and ONH anatomy on these parameters.

## Materials and methods

### Participants

This was a prospective, cross-sectional, observational study approved by the Institutional Review Board of the University of Campinas (approval number 946.742) and conducted in accordance with the Declaration of Helsinki. Healthy subjects aged between 18 and 80 years, of both sexes, were recruited at the Clinics Hospital of the University of Campinas, São Paulo. Participants were either hospital employees or patients’ family members. We aimed to recruit 15 to 20 subjects in each decade of life.

The inclusion criteria were: (1) age between 18 and 80 years-old, (2) best-corrected visual acuity (BCVA) ≥ 20/40, (3) refractive error within ± 6.0 spherical diopters and ≤ 2.0 cylinder diopters, (4) intraocular pressure (IOP) ≤ 21 mmHg, (5) normal clinical eye examination with a normal appearing optic disc (both eyes had to have intact neuroretinal rims and no disc hemorrhage, notch, localized pallor, or cup-to-disc ratio asymmetry > 0.2), and (6) normal visual fields (Glaucoma Hemifield Test, mean deviation and pattern standard deviation within normal limits). Exclusion criteria were: (1) clinically significant vitreoretinal, neuro-ophthalmological or choroidal diseases, (2) uveitis, (3) prior refractive surgery or intraocular surgery, except for non-complicated cataract, (4) subjects with a history of ocular hypertension, angle closure or glaucoma suspect diagnosis in either eye, (5) unreliable visual fields (false positive rate ≥ 15% and false negative rate ≥ 20%) and (6) poor quality OCT images (signal strength < 20). Participants were allowed to repeat the visual field once if the results were unreliable. If both eyes were eligible, one eye was randomly selected as the study eye.

### Procedures

After oral and written informed consent was obtained, participants underwent a complete ophthalmic examination, which included a review of medical history, automated refraction (Topcon AR RM-8000B, Topcon, Tokyo, Japan), best-corrected visual acuity, slit-lamp biomicroscopy, IOP measurement with Goldmann applanation tonometry, gonioscopy, undilated funduscopic examination with a handheld 78 diopter lens, axial length and central corneal thickness measurement (Lenstar LS900, Haag-Streit AG, Koeniz, Switzerland), automated perimetry (SITA standard 24–2, Humphrey Field Analyzer, Carl Zeiss Meditec Inc, Dublin, CA) and OCT (Spectralis, version 6.0.10.0, Heidelberg Engineering, Heidelberg, Germany) imaging. Self-reported race also was recorded. Only individuals who reported being AD, ED or MD (who comprised both European and African ancestry) were included in the study. Participants were examined between November, 2014 and October, 2016.

### Spectral-domain optical coherence tomography

**Fig 1:**
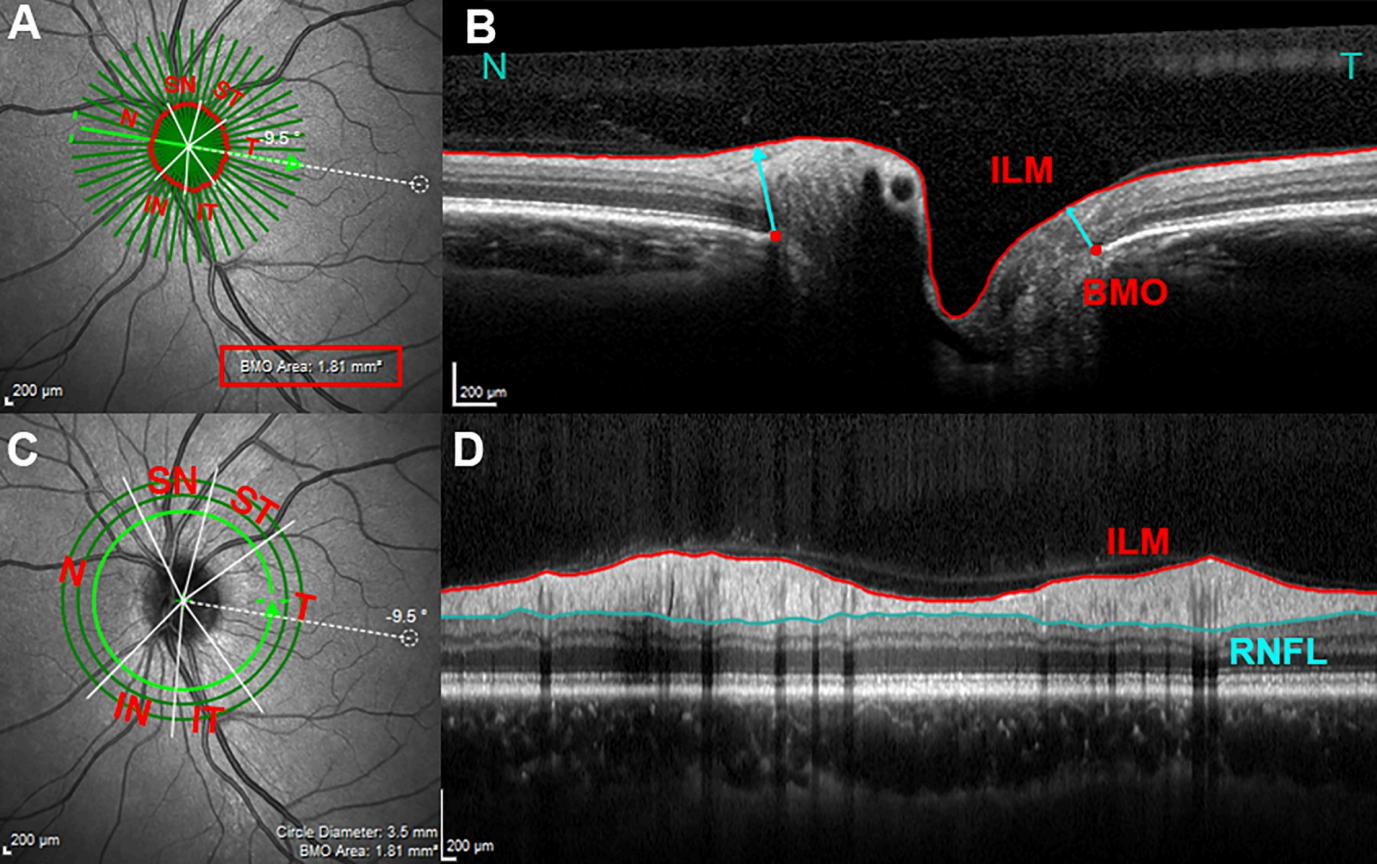
Spectral-domain optical coherence tomography imaging and parameters’ measurements. The infrared image in (A) shows 24 radial B-scans centered on the ONH (dark green radial lines) acquired relative to the orientation of the FoBMO center axis (green arrow connecting the fovea and the BMO center, -9.5° in this subject). In (B), one horizontal radial B scan shows MRW measurements. MRW (cyan arrow) is the minimum distance between BMO (red dots) and ILM (delineated in red). Each B scan yield 2 BMO points and 2 MRW measurements, in this example a nasal (N) and a temporal (T) measurement. The 3D coordinates of each of the 48 BMO points are used to fit a spline which derives a closed curve representing the BMO area (1.81 mm in this disc). In (C) the 3.5 mm diameter circular scan (light green circle) centered on the ONH was used for RNFLT measurements. (D) Is the corresponding circular B-scan showing the ILM (red) and RNFL (cyan) delineations. MRW and RNFLT measurements were averaged and analyzed globally and in 6 sectors: ST(superotemporal), SN (superonasal), IT (inferotemporal), IN (inferonasal), T (temporal), and N (nasal), in relation to the FoBMO center axis.

The ONH and peripapillary RNFL were imaged with a scan speed of 40,000 A-scans/second. The acquisition protocol has been detailed elsewhere [15]. Briefly, 2 scan patterns were obtained: ONH (24 radial scans centered on BMO) and peripapillary scans (3 concentric circle scans of 3.5, 4 and 4.5 mm in diameter). Both scans were acquired relative to its eye-specific FoBMO axis, which was determined before the acquisition. For the 24 radial B-scans, settings were fixed at 15°, 768 A-scans per B-scan, with each B-scan representing an average of 25 scans. For the circle scans, settings were fixed at 3.5, 4 and 4.5 mm diameters centered on the BMO, 768 A-scans, with each circle scan averaged 100 times (**Fig 1**). Only the 3.5 mm circle scan was used for analysis. All participants were imaged by one experienced examiner (C.S.Z.), who also checked all automatic segmentations and manually corrected the BMO, ILM and RNFL segmentations in the 24 radial scans and in the 3.5mm circle scans, when necessary. We chose to have only one examiner since both RNFLT and MRW have shown excellent intra- and interobserver reproducibility on a previous study published by our group [23]. The following parameters were evaluated and automatically generated by the Heidelberg software (version 1.9.13.0): FoBMO Angle, BMO area, MRW and RNFLT.

### Analysis

The following parameters were evaluated: FoBMO Angle, BMO area, MRW and RNFLT. MRW and RNFLT measurements were averaged and analyzed globally and in 6 sectors: four of 40° (superonasal, inferonasal, inferotemporal, superotemporal), one of 90° (temporal), and one of 110° (nasal) [24]. All measurements were regionalized relative to the subjects’ FoBMO axis. Demographic and ocular parameters were compared among groups with one-way analysis of variance (ANOVA). Analysis of covariance (ANCOVA) was conducted to evaluate mean differences in RNFLT and MRW among the three racial groups, adjusting for age and BMO area. The chi-square test was used for categorical variables. We used multivariable linear regression models with interaction terms to evaluate if the effects of co-variables, such as age and BMO area, on MRW or RNFLT. Measurements were adjusted for age and BMO area since previous studies have shown a significant association between both parameters (RNFLT and MRW) and BMO area and age, in normal individuals [15, 16]. We evaluated the relationship between sectorial MRW and RNFLT, adjusted for BMO area, age and race. Partial R^2^ values estimating the relative importance of each predictor were reported [25]. The sectorial age-related loss of MRW and RNFLT was calculated and reported as the percentage loss. Data analysis was performed with the open-source software R [26].

## Results

Among the 304 individuals recruited, 44 were excluded for the following reasons: abnormal or non-reliable visual fields (n = 18), visually significant cataract (BCVA<20/40) (n = 2), diabetic retinopathy (n = 2), epirretinal membrane (n = 1), age-related macular degeneration (n = 1), low quality OCT scan (n = 2), chorioretinal macular scar (n = 1), optic disc hypoplasia (n = 1) and glaucoma suspect (n = 14). Therefore, a total of 260 (85.5%) participants were included in the analysis, of whom 78 (30%) were self-identified as AD, 103 (40%) as ED and 79 (30%) as MD. There were between 17 and 21 ED, 14 and 19 AD and 15 and18 MD participants in each decade group, except for the 8^th^ decade, which included 7 subjects in the ED group, 3 in the AD group and no participants in the MD group.

**Fig 2:**
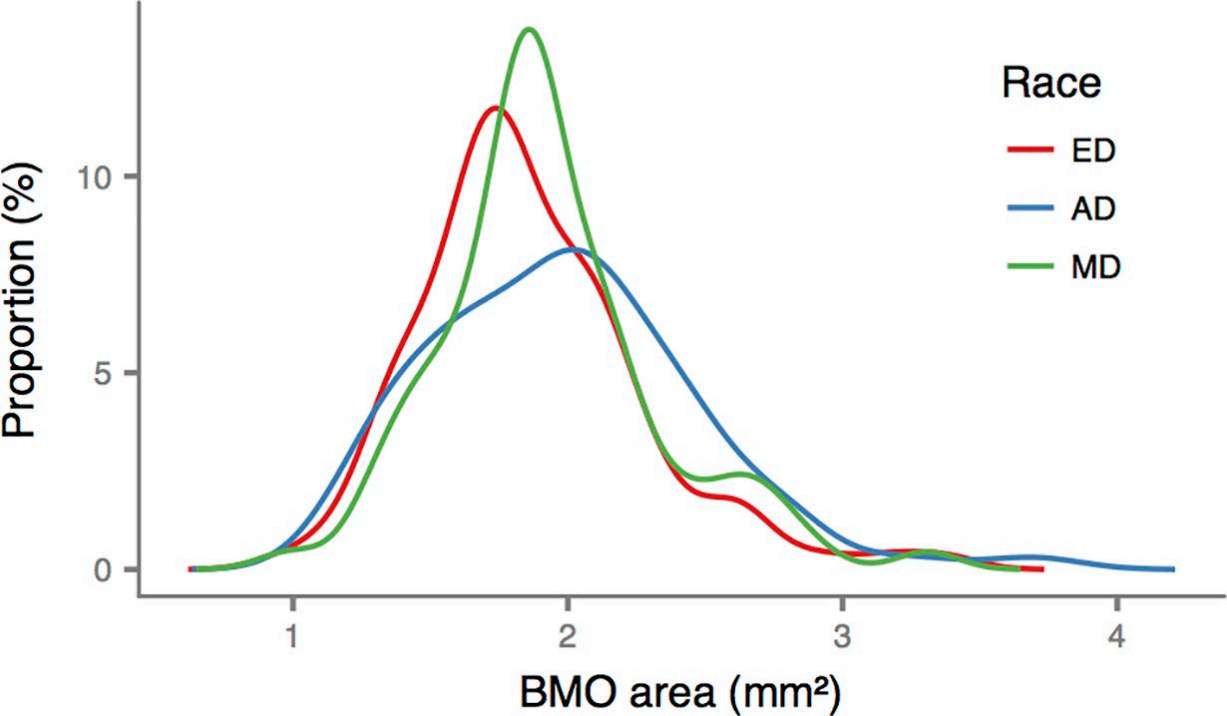
Smoothed histogram showing the distribution of Bruch’s membrane opening (BMO) areas for each race. ED: European Descent; AD: African Descent; MD: Mixed Descent.

**Fig 3:**
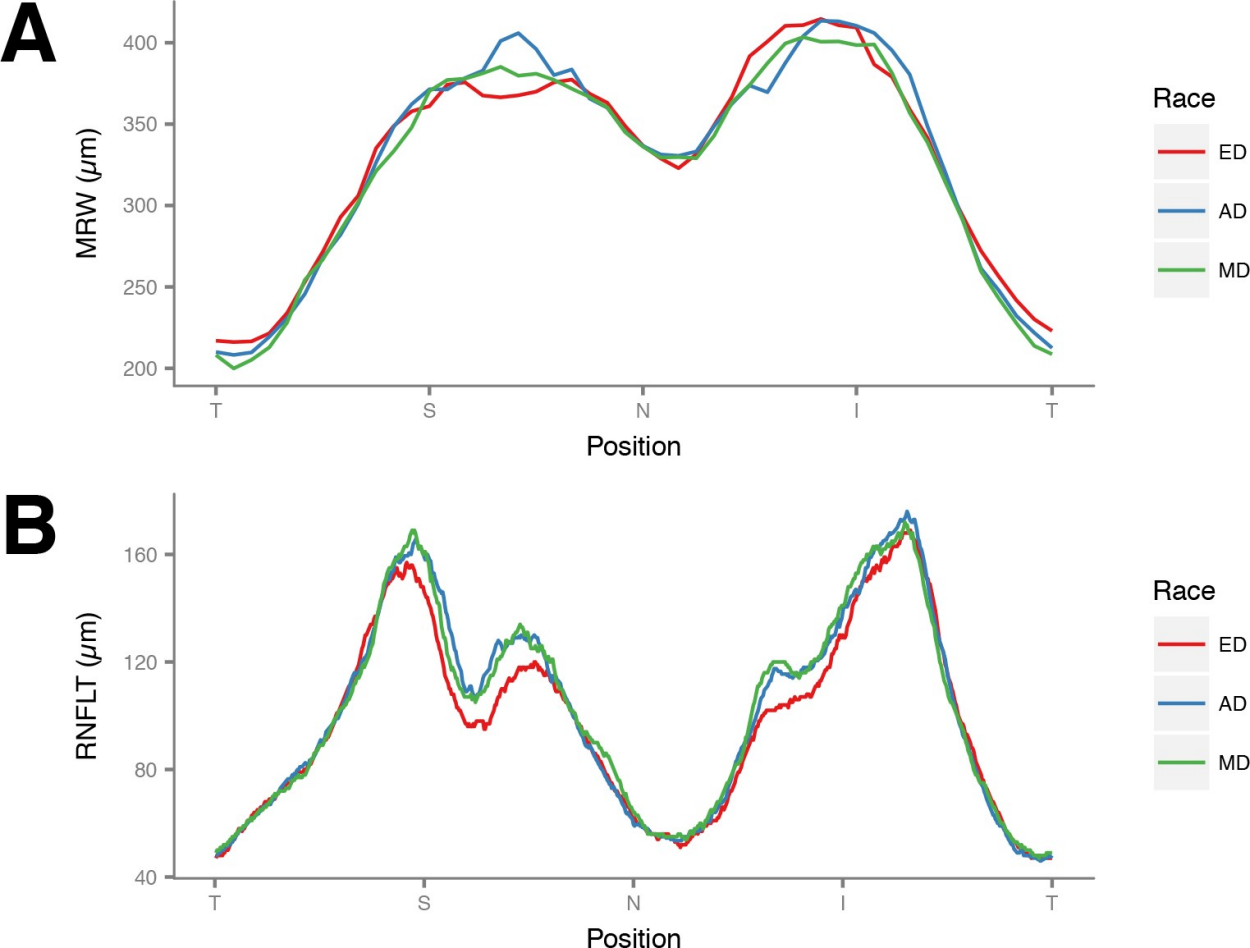
**Graphs showing (A) minimum rim width (MRW) and (B) peripapillary retinal nerve fiber layer thickness (RNFLT) profiles for each race**. ED: European Descent; AD: African Descent; MD: Mixed Descent. T: temporal; S: superior; N: nasal; I: inferior.

Overall, the mean age was 44.9 years old and 60% were female. There were no significant differences among the racial groups in the measured demographic and ocular characteristics (**Table 1**). The BMO areas among the three groups were not significantly different (**Table 1** and **Fig 2**). **Fig 3** shows the MRW and RNFLT profiles for the three groups. There were no significant differences in mean global MRW among the three racial groups (P = 0.63, **Table 2**). Mean global RNFLT was thinner in ED (P = 0.01), but this difference was statistically eliminated after adjusting for BMO area and age (P = 0.07, **Table 3**). In regional analysis, however, after adjusting for BMO area and age, both MRW and RNFLT were greater in AD in the superonasal sector (P = 0.04, P<0.001, respectively) while RNFLT was greater among AD and MD in the inferonasal sector (P = 0.009, **Tables 2 and 3**).

**Table 1:** Clinical characteristics of study subjects.

		All (n = 260)	ED (n = 103)	AD (n = 78)	MD (n = 79)	P*
**Age, years**		44.90 (14.5) [17.8–79.3]	46.49 (15.23) [17.8–78.3]	43.92 (15.12) [18.1–79.3]	43.8 (12.78) [19.7–68.6]	0.36
**Gender F/M, %**		60/40	60/40	63/37	59/41	0.90
**CCT, μm**		531.26 (33.63) [442–635]	535.75 (32.0) [454–635]	526.58 (35.3) [454–596]	530.14 (33.57) [442–609]	0.17
**AXL, mm**		23.46 (0.96) [21.02–27.59]	23.47 (0.99) [21.02–27.59]	23.55 (0.88) [22.01–25.56]	23.36 (0.99) [21.15–26.02]	0.45
**FoBMO (°)**		-6.54 (3.71) [-15.32–3.72]	-6.96 (3.83) [-15.19–2.66]	-5.96 (3.69) [-15.17–2.30]	-6.56 (3.55) [-15.32–3.72]	0.20
**BMO area, mm**^**2**^		1.92 (0.43) [0.98–3.69]	1.86 (0.41) [0.98–3.37]	1.98 (0.48) [1.15–3.69]	1.93 (0.40) [0.99–3.31]	0.16

Values shown are mean (standard deviation) and [range].

ED: European descent; AD: African descent; MD: Mixed descent; CCT: central cornea thickness; AXL: axial length; MRW: minimum rim width; BMO: Bruch’s membrane opening; RNFLT: retinal nerve fiber layer thickness

*ANOVA was used for all variables, except gender, which was analyzed with Chi-square test.

**Table 2:** Comparison of global and sectorial minimum rim width (MRW) measurements by race.

	ED (n = 103)	AD (n = 78)	MD (n = 79)	P*	*P* Adjusted**
**Global**	334.06 (50.15) [242.02–460.84]	335.5 (56.63) [223.69–478.85]	328.12 (50.71) [236.96–487.77]	0.63	0.34
**Temporal**	236.83 (45.44) [130.8–358.37]	235.3 (54.35) [120.05–361.61]	230.41 (44.89) [134.45–335.04]	0.65	0.51
**Superotemporal**	319.88 (52.62) [202.04–447.14]	323.75 (60.01) [155.75–469.12]	314.41 (52.76) [198.77–448.65]	0.56	0.36
**Inferotemporal**	360.17 (60.34) [245.44–574.53]	365.92 (66.58) [221.91–520.98]	352.07 (59.39) [242.98–518.89]	0.37	0.19
**Nasal**	365.86 (61.46) [247.7–518.23]	364.16 (71.15) [232.31–559.57]	360.09 (67.53) [217.94–585.92]	0.84	0.64
**Superonasal**	374.05 (66.74) [221.19–574.36]	391.92 (72.37) [264.43–541.16]	375.57 (66.87) [216.52–513.56]	0.17	**0.04**
**Inferonasal**	413.52 (69.49) [275.88–586.47]	407.09 (74.1) [248.17–604.78]	402.38 (65.00) [285.09–619.11]	0.55	0.45

Values shown are mean (standard deviation) and [range] in μm.

ED: European descent; AD: African descent; MD: Mixed descent

*ANOVA

**ANCOVA to evaluate mean differences in MRW between the three racial groups correcting for age and BMO area.

P values < 0.05 are shown in bold.

**Table 3:** Comparison of global and sectorial retinal nerve fiber layer thickness (RNFLT) measurements by race.

	ED (n = 103)	AD (n = 78)	MD (n = 79)	P*	*P* Adjusted**
**Global**	99.59 (9.57) [81–129]	103.53 (11.59) [74–132]	103.52 (9.64) [65–130]	**0.01**	0.07
**Temporal**	68.2 (10.45) [45 – 101]	68.18 (11.91) [32 – 107]	68.66 (9.53) [46 – 95]	0.94	0.73
**Superotemporal**	128.42 (22.27) [76–172]	128.67 (24.57) [70–195]	128.8 (21.58) [76–175]	0.99	0.82
**Inferotemporal**	154.24 (19.39) [117–212]	157.14 (20.41) [93–211]	155.7 (18.84) [75–198]	0.61	0.95
**Nasal**	82.9 (13.13) [55 – 122]	85.21 (15.57) [48 – 127]	86.47 (11.12) [61–116]	0.18	0.38
**Superonasal**	116.72 (23.83) [66–198]	134.51 (28.07) [83–214]	127.87 (21.6) [74–183]	**<0.001**	**<0.001**
**Inferonasal**	116.52 (23.11) [65–185]	124.12 (21.55) [78–178]	127.81 (22.52) [72–200]	**0.003**	**0.009**

Values shown are mean (standard deviation) and [range] in μm.

ED: European descent; AD: African descent; MD: Mixed descent

*ANOVA

**ANCOVA to evaluate mean differences in RNFLT between the three racial groups correcting for age and BMO area.

P values<0.05 are shown in bold.

**Fig 4:**
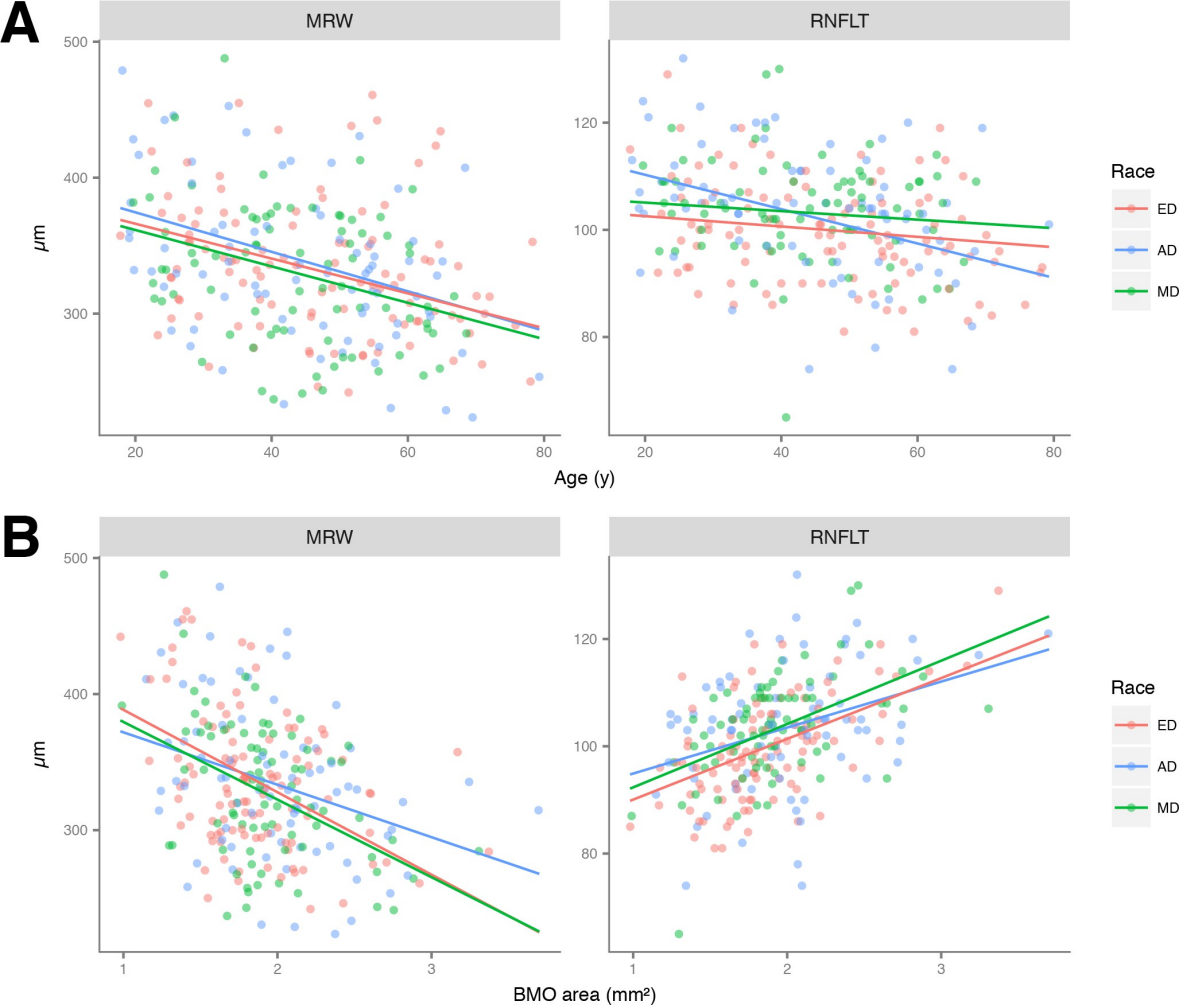
(A) Scatterplots showing the relationship between global minimum rim width (MRW) and age (left) and global peripapillary retinal nerve fiber layer thickness (RNFLT) and age (right), by racial group. Lines represent the coefficients of multivariable regression, adjusted for Bruch’s membrane opening area. (B) Scatterplots showing the relationship between global minimum rim width (MRW) and Bruch’s membrane opening (BMO) area (left) and global peripapillary retinal nerve fiber layer thickness (RNFLT) and BMO area (right) by racial group. Lines represent the coefficients of multivariable regression, adjusted for age. ED: European Descent; AD: African Descent; MD: Mixed Descent.

Global MRW declined significantly with age, after adjusting for BMO area, in AD (-1.45 μm/year; P<0.01 versus 0), the amount by which was not different in ED (-1.28 μm/year; P = 0.71) or MD (-1.34 μm/year; P = 0.83). The rate of global MRW with age was similar in ED and MD (P = 0.91; **Table 4**). The age-related reduction of global RNFLT, adjusted for BMO area, was significant in AD (-0.32 μm/year; P<0.01 versus 0) and was faster than that compared to ED (-0.10 μm/year, P = 0.01) or MD (-0.08 μm/y, P = 0.02). The rate of global RNFLT reduction with age was similar in ED and MD (P = 0.86; **Table 4** and **Fig 4**).

**Table 4:** Multivariable regression models evaluating the relationship between minimum rim width (MRW) and peripapillary retinal nerve fiber layer thickness (RNFLT) and covariates by racial group.

	ß (95% CI)			*P* value		
	AD	ED	MD	AD vs ED	AD vs MD	ED vs MD
**MRW**						
Age* (μm/y)	-1.45 (-2.12, 0.79)	-1.28 (-1.87, -0.70)	-1.34 (-2.12, -0.56)	0.71	0.83	0.91
BMO area** **(**μ**m/mm**^**2**^**)**	-38.51 (-59.28, -17.74)	-60.56 (-82.24, -38.88)	-57.04 (-82.00, -32.08)	0.15	0.26	0.83
RNFLT***(μm/μm)	2.05 (1.20, 2.90)	1.60 (0.67, 2.54)	1.72 (0.71, 2.74)	0.45	0.61	0.85
**RNFLT**						
Age* (μm/y)	-0.32 (-0.45, -0.19)	-0.10 (-0.21, 0.02)	-0.08 (-0.23, 0.07)	**0.01**	**0.02**	0.86
BMO area** **(**μ**m/mm**^**2**^**)**	8.57 (4.48, 12.67)	11.33 (7.05, 15.60)	11.81 (6.88, 16.73)	0.15	0.26	0.83

AD: African descent; ED: European descent; MD: Mixed descent; BMO: Bruch’s membrane opening

*Adjusted for BMO area

**Adjusted for age

*** Adjusted for age and BMO area

P values < 0.05 are shown in bold.

**Fig 5:**
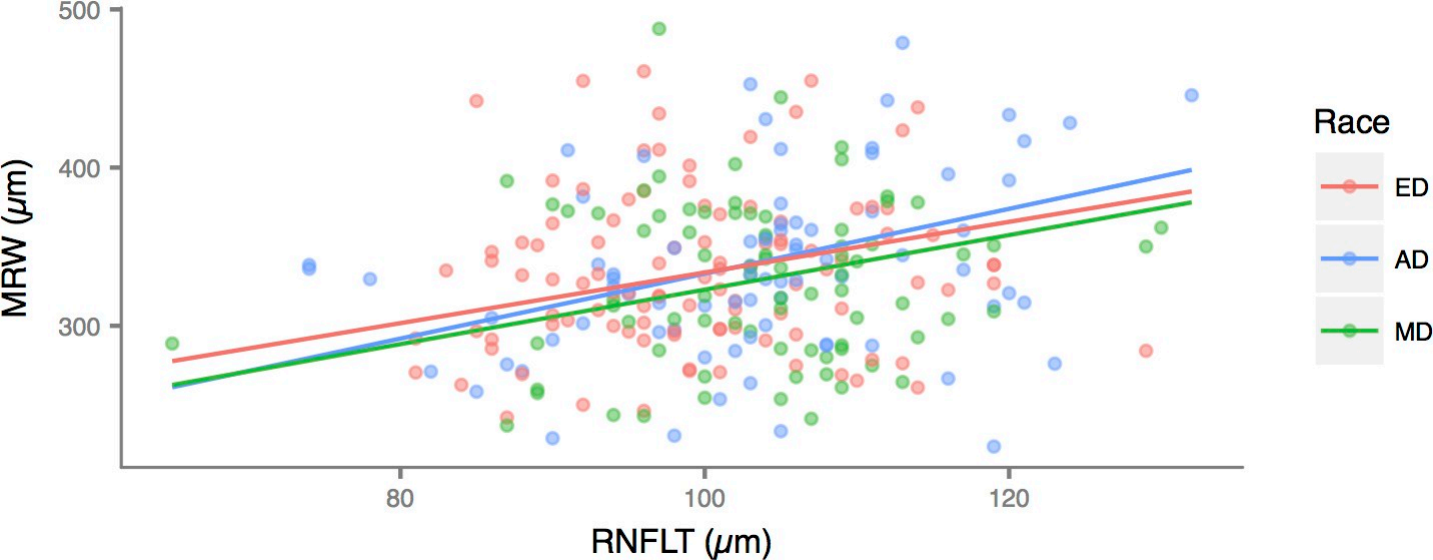
Scatterplot showing the relationship between global minimum rim width (MRW) and peripapillary retinal nerve fiber layer thickness (RNFLT) by racial group. The relationships did not differ among the three groups. ED: European Descent; AD: African Descent; MD: Mixed Descent.

After adjusting for age, global MRW declined significantly with BMO area in AD (-38.51 μm/mm^2^), and this relationship was not different in ED (-60.56 μm/mm^2^, P = 0.15) and MD (-57.04 μm/mm^2^, P = 0.26). The rates of global MRW reduction were also similar between ED and MD (P = 0.83). On the other hand, after adjusting for age, global RNFLT increased significantly with BMO area in AD (8.57 μm/mm^2^) and this was not different in ED (11.33 μm/mm^2,^ P = 0.15) and MD (11.81 μm/mm^2^, P = 0.26). The rates of global RNFLT reduction were also similar between ED and MD (P = 0.83) (**Fig 4**). Global RNFLT increased with MRW, after adjusting for BMO area and age. The adjusted rate was significant in AD (2.05 μm/μm) and did not differ from ED (1.60 μm/μm, P = 0.45) and MD (1.72 μm/μm, P = 0.61). The rates were also similar between ED and MD (P = 0.85) (**Fig 5**).

**Fig 6:**
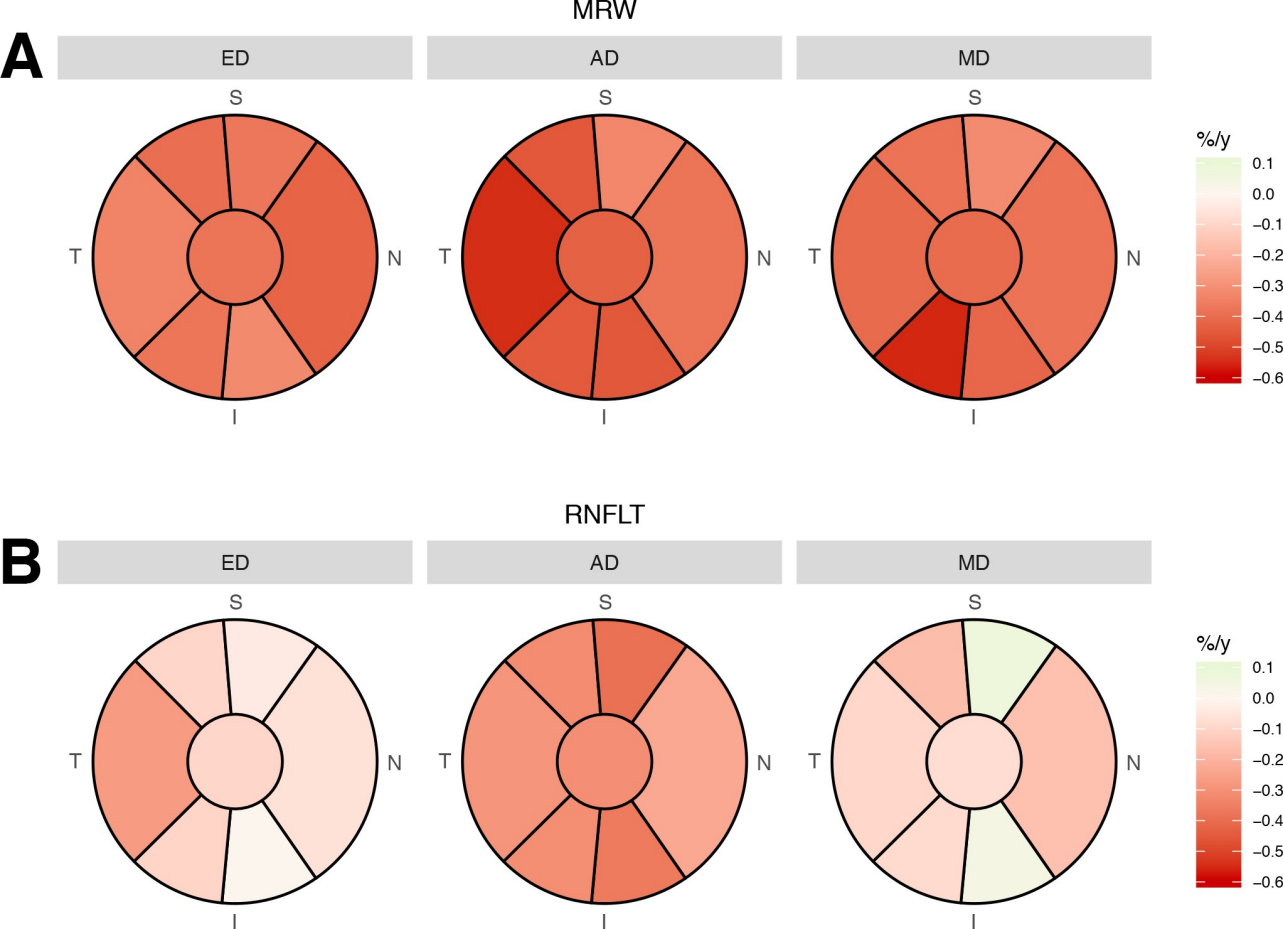
**Graphs showing the sectorial age-related percentage decay (%/y) of (A) mean Bruch’s membrane opening minimum rim width (MRW) and (B) peripapillary retinal nerve fiber layer thickness (RNFLT) globally (central circle) and in 6 sectors**. Mean decay in each sector is color-coded according to the scale in the legend. ED: European Descent; AD: African Descent; MD: Mixed Descent.

Sectorial age-related loss of MRW and RNFLT, adjusted for BMO area, are shown in **Fig 6**. In general, the sectorial age-related percentage decays were faster for MRW than RNFLT, and faster in RNFLT sectors of AD than other races.

The sectorial associations between MRW and RNFLT, adjusted for age, race and BMO area, were variable: it was highest in the inferotemporal sector (R^2^ = 0.17) and lowest in the temporal sector (R^2^ = 0.03). The relative importance of each covariate (RNFLT, age, race and BMO area) in MRW was variable between sectors. While BMO area was the most important predictor of MRW in all sectors, race was the covariable which least explained the variations in this parameter (**Table 5**).

**Table 5:** Multivariable regression model evaluating the relationship between sectorial Minimum Rim Width (MRW) and peripapillary retinal nerve fiber layer thickness (RNFLT), adjusted for Bruch’s membrane opening (BMO) area, age and race. Partial R squared estimating the relative importance of each predictor are given.

Sector	RNFL	Age	BMO Area	Race
**Global** **Temporal**	0.13 0.03	0.11 0.07	0.29 0.08	0.02 0.01
**Superotemporal**	0.15	0.11	0.15	0.01
**Inferotemporal**	0.17	0.11	0.16	0.01
**Nasal**	0.08	0.11	0.30	0.01
**Superonasal**	0.11	0.08	0.22	0.01
**Inferonasal**	0.15	0.12	0.21	0.03

## Discussion

Numerous investigators have characterized racial differences in RNFLT and ONH parameters based on the clinically visible disc margin [17, 18]. Subsequently, there has been debate about the clinical relevance of these differences and the importance of race-specific databases in the evaluation of structural parameters for the diagnosis of glaucoma. Although previous studies did not report an enhanced performance when race-specific databases were used with OCT [27] and HRT [28–30], there is insufficient information about the influence of race on the newer ONH parameters based on BMO.

In this study, we evaluated ED, AD and MD individuals from Brazil and found no significant differences in global MRW and RNFLT among the three racial subgroups included in the study, with the exception that regionally, MRW was thicker in AD in the superonasal sector. Rhodes and colleagues recently compared MRW measurements between AD and ED in a US population and found no significant differences, both globally and regionally, between the two groups [16].

Regarding RNFLT, the three groups had similar global and regional values, except in the superonasal and inferonasal sectors, in which ED had statistically thinner RNFL measurements, even after adjusting for age and BMO area. Other authors have also shown similar global RNFLT between AD and ED, when adjusted for BMO area and when data was acquired and regionalized according to the FoBMO [16]. However, they have also reported significantly thinner temporal RNFLT in AD when compared to ED. In a study with RTVue, Girkin and colleagues also showed that AD from the US had significantly reduced RNFL thickness in the temporal sector and greater RNFL thickness in the superior and inferior regions compared with ED, after adjusting for age and disc area [18]. This previous study regionalized MRW and RNFLT measurements in four quadrants (superior, inferior, nasal and temporal) that were assigned relative to the horizontal axis of the acquired image frame. The location of these regions thus varies in each study eye. In the present study, we regionalized our data in six sectors that were consistently assigned in each study eye, relative to the axis connecting the fovea to BMO center. These differences in data regionalization may account for differences between our findings and previously published data.

In our study, the regional MRW and RNFLT distribution around BMO followed a similar pattern in AD, ED and MD individuals, confirming Rhodes et al findings for AD and ED from the US [16]. Also, similar to what has been previously described in other populations[14–16], the FoBMO angle also varied widely among Brazilian individuals, irrespective of race.

Previous studies in the US have consistently shown that AD have larger optic discs areas when compared to ED, when evaluated in postmortem measurements [31], disc photos [32], OCT [18, 33] and HRT [34–36]. In all these studies, optic disc area was measured based on the clinically visible optic disc margin. However, recently, it has also been shown that eyes of AD from the US have larger BMO areas than ED eyes, in contrast to the findings in our study. As mentioned before, Brazilian ADs come from different African territories compared to US ADs [20–22]. On the other hand, it is also possible that the genetic admixture of the Brazilian population contributed to a more homogeneous ONH phenotype across the racial groups [37, 38]. Finally, these discrepancies may be explained by inaccuracies of the self-reported nature of race definitions employed in all these studies.

Both MRW and RNFLT were correlated with BMO area, which was expected, since there is consistent evidence showing a decrease in MRW with an increase in BMO area [15, 16]. RNFLT in our study, on the other hand, increases significantly with an increase in BMO area. The underlying reasons for this are not entirely clear. One possible explanation is that RNFLT measurements are made closer to the BMO when a fixed (3.5 mm) circular scan is used in an eye with a large BMO area [39]. Another possible explanation is that individuals with large BMO areas may have a greater number of ganglion cells axons, as suggested in histologic studies [31, 40]. Finally, it is possible that eyes with larger BMO areas may have greater amounts of non-axonal components (i.e. glia).

In the present study, all racial groups showed similar rates of MRW decay with age, but AD had significantly higher rates of global RNFLT decay, compared to the other racial groups, a finding that has not been reported in the US population. Rhodes and colleagues described similar rates of MRW and RNFLT decay with age between ED and AD from the US [16], but the specific rates (in μm per year) were not reported. The higher rates of RNFLT decline with age in Brazilian AD eyes, and its underlying causes, may separately contribute to the higher prevalence [41–44] and earlier onset of glaucoma [45–47] in AD eyes. Although most of the population-based studies data have been reported in AD subjects from the US population, there is some evidence of higher prevalence of glaucoma among AD Brazilians[48]. The earlier onset of glaucoma in AD individuals is thought to be the result of a complex interaction of factors, including intraocular pressure, ONH and ocular biomechanics [46]. It is possible that the material properties of the ONH connective tissues of AD individuals differ from ED, leading to greater susceptibility to damage and, subsequently, to greater loss of ganglion cells with age. Regarding the age-related decay of global MRW and RNFLT in ED from the US, Canada and Europe, Chauhan and colleagues reported yearly rates of -1.34 and -0.21 μm/year, respectively [15]. The yearly rate of MRW decay was similar in our ED group, but the yearly decay of RNFLT was lower than what has been reported (-1.28 and -0.10 μm/year, respectively). However, it is important to note that the decay rates, in both studies, were highly variable among individuals (with large CIs), much more than the mean differences between our ED group and the ED group from the US.

Although statistically significant, the correlation between global MRW and global RNFLT was not strong, and was also variable by sector, after correction for BMO area and age. Although this finding is somewhat surprising, since it is expected for both the neuroretinal rim and the peripapillary RNFL to be constituted of the same retinal ganglion cells axons, it may be that differences in the non-axonal constituents of MRW and RNFLT, such as glial tissues and vessels, contribute to a lack of better correlation between these two parameters [49–51]. It is also possible that the spatial correlation between the RNFL and the ONH rim is not perfect and, therefore, the number of axons in the peripapillary retina in a given sector may not be the same number of axons that constitute the ONH rim in that same sector. The correlation between these two parameters seems to be more complex than expected. Chauhan and colleagues reported similar findings[15]. Patel and colleagues also have already reported a nonlinear relationship between them [52].

There are some limitations in our study. Only few individuals over the age of 70 were included in the study, especially in the MD and AD groups, which could impact the differences in age-related MRW and RNFLT decay rates across racial groups. In addition, the self-reported nature of the racial definition is also a source of error because self-reported race may not truly correspond to the real genetic composition of individuals [38, 53–56]. However, even the definition of race based on genetic findings is controversial [57], and the majority of population-based studies rely on self-reported racial definitions [18, 58].

While we described differences in some of the mean MRW and RNFLT sectorial measurements in AD, MD and ED from Brazil, these differences were small compared to the normal variations in ONH structure within each race. Therefore, there is a suggestion that a race-specific normative database may not be necessary. It may well be that non-race-specific databases with larger number of individuals will provide narrower 95% CIs than race-specific ones, actually increasing diagnostic accuracy when used compared to race-specific databases. In clinical practice, it means that when evaluating a glaucoma suspect, the comparison between patient’s data to a race-specific normative database may not add to the diagnostic capability of the method. However, this may not be true for detection of progression. Age-related thinning of the RNFLT was significantly higher in the AD subgroup, which warrants further studies.

## Supporting information

S1 File (CSV)

S2 File (CSV)

S3 File (CSV)
